# Structure Elucidation and *in Vitro* Toxicity of New Azaspiracids Isolated from the Marine Dinoflagellate *Azadinium poporum*

**DOI:** 10.3390/md13116687

**Published:** 2015-10-30

**Authors:** Bernd Krock, Urban Tillmann, Éric Potvin, Hae Jin Jeong, Wolfgang Drebing, Jane Kilcoyne, Ahmed Al-Jorani, Michael J. Twiner, Qun Göthel, Matthias Köck

**Affiliations:** 1 Alfred-Wegener-Institut, Helmholtz-Zentrum für Polar- und Meeresforschung, Am Handelshafen 12, Bremerhaven 27570, Germany; E-Mails: urban.tillmann@awi.de (U.T.); wolfgang.drebing@awi.de (W.D.); qgothel@jausoft.com (Q.G.); 2 Division of Polar Ocean Environment, Korea Polar Research Institute, Incheon 406-840, Korea; E-Mail: ericpotvin@kopri.re.kr; 3 School of Earth and Environmental Science, Seoul National University, Seoul 151-747, Korea; E-Mail: hjjeong@snu.ac.kr; 4 Marine Institute, Rinville, Oranmore, Co. Galway, H91 R673, Ireland; E-Mail: jane.kilcoyne@marine.ie; 5 Department of Natural Sciences, University of Michigan, Dearborn, MI 48202, USA; E-Mails: ahmed_aljorany@yahoo.com (A.A.-J.); mtwiner@med.wayne.edu (M.J.T.)

**Keywords:** shellfish poisoning, lipophilic marine biotoxins, NMR

## Abstract

Two strains of *Azadinium poporum*, one from the Korean West coast and the other from the North Sea, were mass cultured for isolation of new azaspiracids. Approximately 0.9 mg of pure AZA-36 (**1**) and 1.3 mg of pure AZA-37 (**2**) were isolated from the Korean (870 L) and North Sea (120 L) strains, respectively. The structures were determined to be 3-hydroxy-8-methyl-39-demethyl-azaspiracid-1 (**1**) and 3-hydroxy-7,8-dihydro-39-demethyl-azaspiracid-1 (**2**) by ^1^H- and ^13^C-NMR. Using the Jurkat T lymphocyte cell toxicity assay, (**1**) and (**2**) were found to be 6- and 3-fold less toxic than AZA-1, respectively.

## 1. Introduction

The first azaspiracid poisoning (AZP) event was recorded in 1995 after eight people experienced severe gastrointestinal disorders following consumption of mussels originating from the Irish West Coast. Symptoms were typical of diarrhetic shellfish poisoning (DSP). Even though the mouse bioassay for DSP toxicity was also strongly positive, no DSP toxins could be detected at elevated levels and thus could not account for the observed severe intoxications [1].

In 1998, the causative compound was isolated and characterized from shellfish and subsequently named azaspiracid-1 (AZA-1) according to its structural properties [2]. A few years later, the synthesis of AZA-1 led to its structural revision [3]. AZAs are polyketides consisting of highly hydroxylated linear carbon chains that are cyclized at several points by formation of ether bridges. In addition, AZAs typically contain a six-membered cyclic imine ring [2,3].

After AZAs were confirmed to be responsible for gastrointestinal disorders associated with the consumption of seafood, further AZP incidents were reported from France and Italy [4]. To date, there are no systematic global surveys on the geographical distribution of AZAs but there are several reports of shellfish contaminated with AZAs, such as from the East coast of England and along the Norwegian West Coast [5], Portugal [6], Morocco [7], Chile [8], Japan [9] and China [10]. In contrast, monitoring of lipophilic phycotoxins in mussels from the White Sea (Russia) did not indicate the presence of AZAs [11]. More than two dozen naturally occurring structural variants of AZA-1 have been described [12]. Although most research has focused on AZA-1 as the first discovered and most accessible AZA analogue, other structural variants have been isolated and characterized from shellfish [13,14,15,16].

In 2007, an AZA-producing organism was isolated [17] and identified as *Azadinium spinosum* [18]. Bulk culturing of this organism was performed with subsequent isolation of AZA-1, -2 [19], -33 and -34 and description of AZA-35 [20]. In addition to *A. spinosum*, a number of new species of the genus *Azadinium* were described from the North Sea: *A. obesum* [21], *A. poporum* [22], and *A. polongum* [23]. A species designated as *A.* cf. *spinosum* was reported to form blooms at the Argentinean shelf [24], *A. dexteroporum* was described from the Mediterranean [25], *A. dalianense* from the coast of China [26], and more recently three new species have been described from the Irminger Sea (North Atlantic) [27]. In addition to the European strains of *A. poporum*, there also exists a strain from Korean coastal waters [28] and a number of strains from China [29]. Based on minor differences in shape of the 3′ plate and variability in ribosomal DNA sequences between the Korean and the European strains, the Korean isolate was initially designated as *A.* cf. *poporum*. However, this trait was subsequently shown to vary among and within other Asian *A. poporum* strains [29], which supports the notion that *A. poporum* from Europe and Asia, despite their differences in ITS and 28S gene sequences [29], are conspecific. Most species of the genus *Azadinium* were originally described as non-AZA producers [21,22,23,26]. However, in various strains of *A. poporum* we recently detected compounds that show fragmentation patterns with high similarities to those of AZAs. Based on collision induced dissociation (CID) spectra and high resolution mass spectral analysis, putative molecular structures for these new toxins were proposed [30].

In this study, the structures of these compounds were verified using nuclear magnetic resonance (NMR) spectroscopy and their *in vitro* potencies were obtained using the Jurkat T lymphocyte cell assay.

## 2. Results

### 2.1. Purification of AZA-36 (**1**) and AZA-37 (**2**)

We purified AZA-36 (**1**) and AZA-37 (**2**) from two strains of *A. poporum*, one from the Western Pacific [28] and the other from the North Sea [22] using procedures previously described [19]. Four isolation steps were required to purify (**1**) from the HP20 extracts (Table 1). For (**1**) a relatively high amount of culture (870 L) was needed for a final yield of 0.89 mg, because the toxin cell quota of the Korean strain of *A. poporum* with ~2 fg·cell^−1^ was relatively low [30].

**Table 1:** Batch summary table for purification of AZA-36 (**1**).

Step No	Step	AZA-36 [mg]	Weight [g]	Purity [%] ^†^
	HP20 resin extract	1.30	8.30	<0.1
1	Ethyl acetate partitioning	1.20	0.74	0.2
2	Silica gel	1.05	0.18	0.6
3	Flash (phenyl-hexyl)	0.92	<0.01	40.0
4	Prep HPLC (C18)	0.89	-	> 95
	% Recovery (steps 1−4)	69		

^†^ Based on w/w.

In contrast to the Korean strain, the North Sea strain had an approximately tenfold (**2**) cell quota of ~20 fg·cell^−1^ [30] and thus only 120 L of culture were needed to give a final amount of 1.28 mg pure (**2**) (Table 2).

**Table 2:** Batch summary table for purification of AZA-37 (**2**).

Step No	Step	AZA-37 [mg]	Weight [g]	Purity [%]^†^
	HP20 resin extract	1.70	1.03	0.2
1	Silica gel	1.60	0.25	0.6
2	Flash (phenyl-hexyl)	1.57	0.01	20.0
3	Prep HPLC (C18)	1.28	-	>95
	% Recovery (steps 1−3)	75		

^†^ Based on w/w.

### 2.2. Structure Elucidation of AZA-36 (**1**) and AZA-37 (**2**)

**Figure 1:**
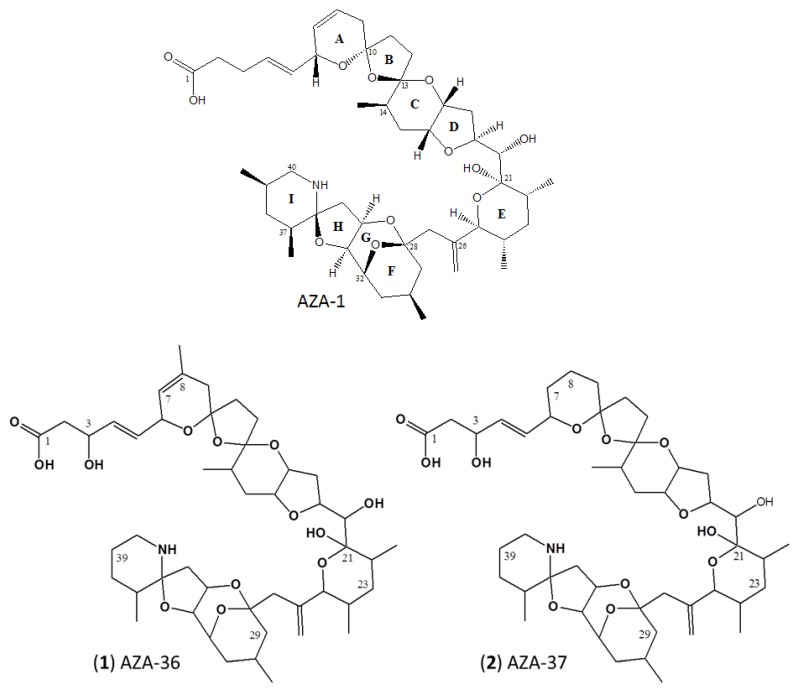
Structures of AZA-1 (top), AZA-36 (**1**) (bottom, left), and AZA-37 (**2**) (bottom, right).

The chemical shifts (^1^H and ^13^C) of (**1**) and (**2**) are listed in Table 3. The corresponding spectra are given as supplementary material. Compared to AZA-1, the ^1^H-NMR spectrum of (**1**) (Table 3) showed an extra oxymethine signal at δ 4.43, an allylic methyl signal at δ 1.70, and the loss of the 39-methyl signal. The ^1^H,^1^H-COSY correlations between H-2/H-3 and H-3/H-4 confirmed the neighborhood of H-2 and H-4 to the oxymethine (H-3). The ^13^C signal of C-3 at δ 71.4 indicated that C-3 of (**1**) was substituted by a hydroxyl group. The ^1^H,^13^C-HMBC spectrum showed correlations from H-47 (δ 1.70) to C-7 (123.4), C-8 (132.1), and C-9 (41.1), and the ^1^H,^1^H-COSY spectrum showed correlations between H-47/H-7 (δ 5.36) and H-47/H-6 (δ 4.79). These correlations allowed the assignment of the new allylic methyl group. Moreover, the methyl group at C-39 was substituted by a proton according to the ^1^H,^1^H-COSY spectrum. The other subunits in (**1**) were assigned on the basis of the 1D and 2D NMR experiments. Based on these results, the structure of AZA-36 was deduced to be 3-hydroxy-8-methyl-39-demethyl-azaspiracid-1 (**1**), as shown in Figure 1.

**Table 3:** ^1^H- and ^13^C-NMR chemical shifts of AZA-36 (**1**) and AZA-37 (**2**).

Atom No.	AZA-36			AZA-37		
	δ_C_	δ_H_		δ_C_	δ_H_	
1	180.3 ^a^	-	-	180.3 ^a^	-	-
2	46.0	2.34	2.39	46.1	2.33	-
3	71.4	4.43	-	71.4	4.39	-
4	135.5	5.75	-	134.6	5.70	-
5	132.1	5.64	-	133.1	5.65	-
6	72.8	4.79	-	73.3	4.35	-
7	123.4	5.36	-	38.4	1.43	1.87
8	132.1	-	-	22.2	1.70	1.77
9	41.1	2.00	2.44	36.6	1.70	1.83
10	108.3	-	-	109.1	-	-
11	34.0	1.71	2.33	33.9	1.69	2.33
12	38.3	1.99	2.18	32.8	1.83	2.03
13	112.2	-	-	111.8	-	-
14	32.1	2.02	-	31.8	2.01	-
15	33.3	1.76	1.86	33.5	1.76	1.87
16	78.9	3.91	-	78.9	3.94	-
17	74.2	4.23	-	74.3	4.29	-
18	37.5	1.99	2.06	37.6	2.00	2.07
19	79.9	4.43	-	79.9	4.44	-
20	77.5	3.90	-	77.4	3.93	-
21	101.1	-	-	101.0	-	-
22	37.8	2.06	-	37.6	2.07	-
23	39.1	1.44	1.44	39.1	1.43	1.43
24	43.0	1.35	-	43.0	1.35	-
25	80.4	4.00	-	80.3	4.00	-
26	149.0	-	-	149.0	-	-
27	50.4	2.26	2.43	50.4	2.25	2.42
28	99.4	-	-	99.4	-	-
29	45.0	1.37	2.05	45.0	1.36	2.05
30	27.2	2.23	-	27.2	2.23	-
31	36.2	1.52	1.83	36.1	1.52	1.84
32	73.7	4.37	-	73.7	4.37	-
33	82.1	4.05	-	82.1	4.05	-
34	75.7	4.99	-	75.7	5.00	-
35	42.8	2.49	2.61	42.7	2.49	2.60
36	98.0	-	-	98.0	-	-
37	37.5	1.99	-	36.7	1.98	-
38	29.8	1.61	1.68	29.7	1.63	1.67
39	23.8	1.70	1.81	23.8	1.70	-
40	41.3	2.98	3.17	41.2	2.99	3.17
41	17.5	0.94	-	17.5	0.90	-
42	17.2	0.92	-	17.2	0.92	-
43	18.9	0.84	-	18.9	0.84	-
44	117.8	5.16	5.33	117.8	5.15	5.33
45	24.3	0.96	-	24.3	0.96	-
46	16.4	0.98	-	16.4	0.97	-
47	23.0	1.70	-	-	-	-

^a^ Chemical shift value obtained from the ^1^H,^13^C-HMBC spectrum because the signal was not observed in the 1D ^13^C spectrum.

The ^13^C-NMR spectrum of (**2**) showed four olefinic carbons in total, which are two less compared to AZA-1. After examination of the NMR spectra, it was concluded that the four olefinic carbons of (**2**) (C-4, C-5, C-26, and C-44) remained the same as in AZA-1. Therefore, the last double bond of AZA-1 at C-7/C-8 is a single bond in (**2**). Moreover, the new oxymethine signal at δ 4.39 and loss of the 39-Me signal were also observed from the ^1^H-NMR spectrum. These data suggested the same hydroxylated carbon C-3 and the proton substituted methyl group at C-39 in (**1**). Therefore, the structure of AZA-37 was confirmed to be 3-hydroxy-7,8-dihydro-39-demethyl-azaspiracid-1 (**2**), as shown in Figure 1.

### 2.3. Cytotoxicity

**Figure 2:**
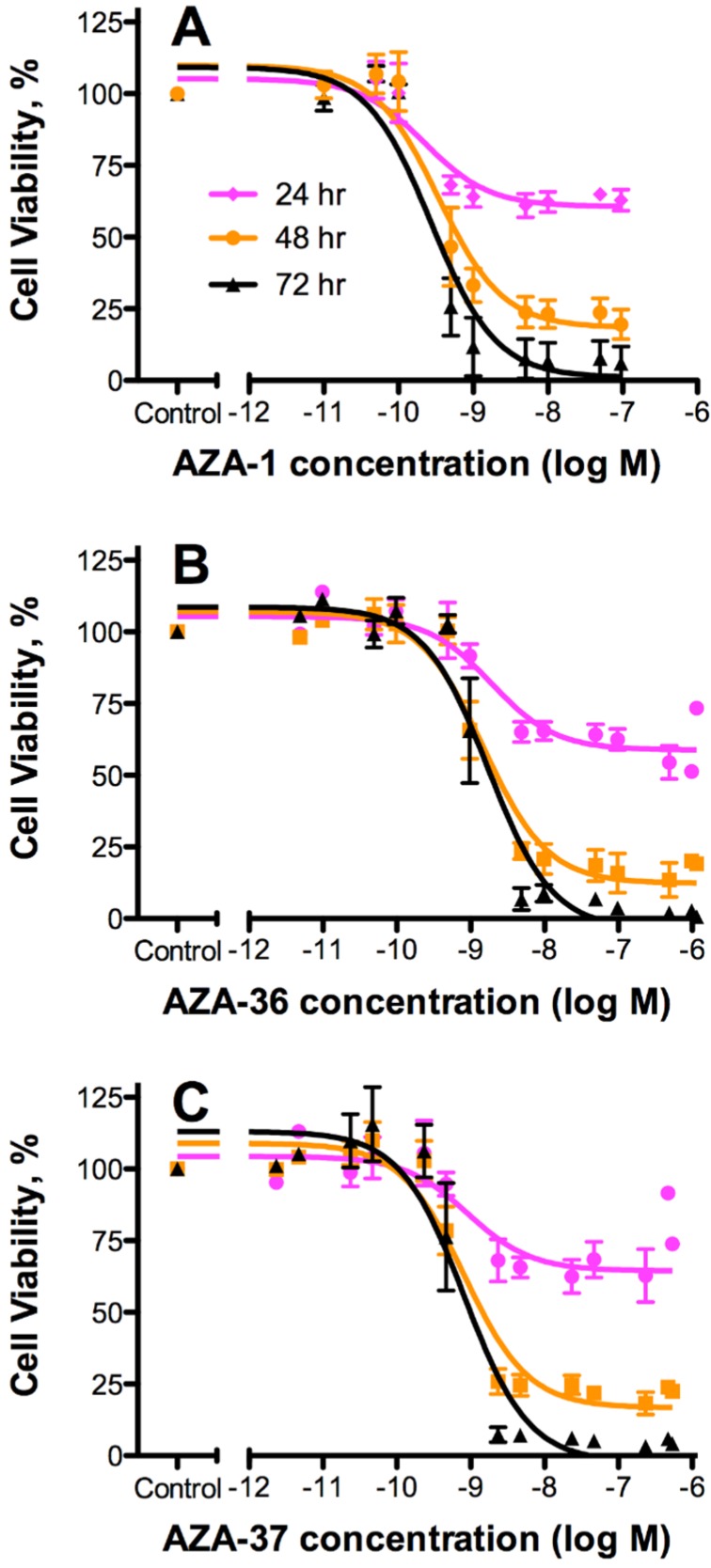
Effect on T lymphocyte cell viability. Jurkat T cells exposed to various concentrations of (**A**) AZA-1; (**B**) AZA-36 (**1**); and (**C**) AZA-37 (**2**) for 24, 48, or 72 h and viability assessed using the MTS assay. All data (mean ± SE; *n* = 4) were normalized to the control (10% methanol vehicle). Non-linear, three parameter dose-response (variable slope) analysis was performed and EC_50_ values were calculated (Table 4).

The toxicity of (**1**) and (**2**) was assessed using the Jurkat T lymphocyte cell assay. In order to compare this data to the known toxicities of other AZAs, AZA-1 was included in this assay and individual toxicities were expressed as relative potencies. Similar to AZA-1, (**1**) and (**2**) elicited concentration- and time-dependent cytotoxic effects towards T lymphocytes but with variable potencies, clearly seen from the dose-response curves of the three compounds (Figure 2). The cytotoxic effects induced by AZA-1 are consistent with previously published studies [31,32,33].

The steep slopes illustrate the narrow toxicological window and the step-wise decrease in viability with longer exposure times (at the higher concentrations) may represent the irreversible nature of the AZAs [34]. Compound (**1**) was found to be the least toxic with a relative (to AZA-1) toxicity of 16% (mean EC_50_:1.70 nM) while (**2**) had a relative toxicity of 33% (mean EC_50_: 0.85 nM) (Table 4).

**Table 4:** Calculated EC_50_ values (with 95% confidence intervals) and relative potencies for AZA-1, AZA-36 (**1**), and AZA-37 (**2**) based on a T lymphocyte cytotoxicity assay.

AZA Analogue	24 h		48 h		72 h		Mean EC_50_ [nM]	Rel. Pot.
	EC_50_ [nM]	95% CI	EC_50_ [nM]	95% CI	EC_50_ [nM]	95% CI		
AZA-1	0.22	0.10–0.49	0.34	0.18–0.65	0.27	0.14–0.52	0.28	1
AZA-36* (**1**)	1.9	0.89–4.1	1.5	0.92–2.4	1.7	0.92–3.2	1.70	0.16
AZA-37 (**2**)	0.91	0.27–3.1	0.82	0.51–1.3	0.82	0.46–1.5	0.85	0.33

* Data corrected for 97% purity.

## 3. Discussion

For many years, the causative organism responsible for the production of AZAs went unidentified. Since the discovery of *A. spinosum* in 2007, many new species have been identified. However, production of AZAs and the diversity and uniqueness of the various analogues has not yet been fully characterized for some of the recently identified *Azadinium* species. In the original species description, *A. poporum* was considered as non-toxic [22], but recent research showed that this dinoflagellate is capable of producing AZAs [30]. However, initial mass spectral data indicated that some of the AZAs produced by *A. poporum* were structurally unique from previously reported analogues by having a modification of the nitrogen-containing I-ring of the molecule [29,30,35]. The sum formula of (**1**) had previously been determined by high resolution mass spectrometry as C_47_H_72_NO_13_ and the elemental composition of (**2**) had been determined as C_46_H_72_NO_13_ [30]. Mass spectral interpretations of collision induced dissociation spectra allowed for the above mentioned structural predictions. However, these predictions are limited by the fact that mass spectrometry only yields the *m*/*z* ratios of the fragments. Thus, mass spectrometry cannot differentiate between isomeric partial structures and full structural elucidation must be achieved by NMR. However, unlike mass spectrometry, NMR requires pure compounds in relatively high amounts (typically between 100 and 1000 µg). For this reason, we purified (**1**) and (**2**) from two strains of *A. poporum*, one from the western Pacific [28] and the other from the North Sea [22]. The standard procedure of extracting harvested cells with organic solvents results in extracts contaminated with a high proportion of matrix compounds, which require many purification steps. In the current study, the extraction protocol included acetone for cell lysis followed by the (SPATT) technique [36] for the extraction of AZAs. This approach was used previously by Jauffrais *et al*. [19] to harvest AZAs from the permeate and non-retentate phases following bulk culturing of *A. spinosum* in photobioreactors. The procedure does not require centrifugation or cell filtration steps, which makes it much more feasible. Only four isolation steps were required to purify (**1**) from the HP20 extracts. In contrast, for the isolation of (**2**) only three purification steps were necessary and the ethyl acetate-water liquid-liquid partitioning used in the purification of (**1**) could be omitted. Accordingly, (**2**) was purified by silica chromatography, flash chromatography, and preparative reversed phase chromatography (Table 2).

### 3.1. Structure Elucidation of AZA-36 (**1**) and AZA-37 (**2**)

The results of the NMR investigation did not entirely confirm the originally proposed structures of (**1**) and (**2**) which were solely based on mass spectral experiments. The structural element that was confirmed is the 39-demethylation of the I-ring in both compounds in comparison to the parent compound AZA-1 (Figure 1). However, the NMR measurements of this study revealed that the hydroxylation site of both (**1**) and (**2**) is located at C-3 instead of the initially proposed C-2 position. In addition, the methyl group of the side chain/A-ring fragment of (**1**) is now known to be located at the C-8 position (instead of the initially proposed C-3 position), a feature shared by both (**1**) and AZA-2. Thus, we could unambiguously assign the structure of 3-hydroxy-8-methyl-39-demethyl-azaspiracid-1 to (**1**).

The structural elements of (**2**), which could be confirmed by NMR, are the above-mentioned 39-demethylation and additionally the 7,8-dihydration. However, the originally suggested 2-hydroxylation is now known to be at the C-3 position and the 3-methylation at C-14 in the C-ring. Collectively, (**2**) is structurally elucidated as 3-hydroxy-7,8-dihydro-39-demethyl-azaspiracid-1 (Figure 1). It is noteworthy that both compounds, with the exception of the 39-demethylation, only show modifications in the side chain and A-ring of the molecule, which is the putative biosynthetic end of AZAs [37].

### 3.2. Cytotoxicity

Our cytotoxicity assays indicated that (**1**) and (**2**) are 6- and 3-fold less toxic than AZA-1 respectively. Both, (**1**) and (**2**) differ from AZA-1 in the lack of the 39-methyl group and in the additional 3-hydroxyl group. Most likely one of the two or even both structural elements seem to have an influence on the toxic potency in the T lymphocyte cell model system. Using the same cytotoxicity method with T lymphocyte cells, the relative cytotoxic potencies of naturally isolated AZA-1, AZA-2, and AZA-3 towards T lymphocytes was AZA-2 > AZA-3 > AZA-1 [31] strongly suggesting that methylation at the C-8 and C-22 positions play a role in determining the toxicity. Although the toxicophore of the AZA molecule has not yet been identified, the A/B/C ring region of the AZA molecule is highly important for maintained cytotoxicity. AZA-33 with an *m*/*z* of 716 (AZA-1 missing A/B/C rings) was significantly less potent than AZA-1 (~5-fold), whereas AZA-34 with an *m*/*z* of 816 (AZA-1 missing C4/C5 alkene) was 5.5-fold more potent than AZA-1 [20]. Similarly, an AZA-1 isomer (37-*epi*-AZA-1) was 5.1-fold more potent than AZA-1 [38]. Semi-synthetic hydrogenated AZA-1 analogues, 4,5-dihydro-AZA-1 and 4,5,7,8-tetrahydro-AZA-1, were also tested. Both compounds were found to be approximately equipotent to AZA-1 [32] suggesting that the C4/C5 alkene and the C7/C8 olefin bonds are not necessary for toxicological activity.

## 4. Experimental Section

### 4.1. Reagents

Acetone and methanol used for cell lysis and resin preconditioning were HPLC grade, purchased from J.T. Baker (Center Valley, PA, USA). The anion exchange resin (Diaion^®^ HP20) was from Supelco^®^ (Steinheim, Germany). All other solvents (pestican grade) were purchased from Labscan (Dublin, Ireland). Sodium chloride (>99%), triethylamine (99%), ammonium acetate (>97%), ammonium formate (reagent grade), formic acid (>98%), and silica gel (10–40 µm, type H) were purchased from Sigma-Aldrich (Steinheim, Germany). Luna Phenyl-Hexyl (15 µm) was from Phenomenex (Cheshire, UK). AZA-1 certified reference material (CRM) was obtained from the NRC, Certified Reference Material Program (Halifax, NS, Canada).

### 4.2. Cell Culture and Extraction

A total of 870 L *A. poporum* strain HJ-2010 (hereafter named “Korea”) isolated as described in [28] from Shiwha Bay, a highly eutrophic bay from Korea (37°18′ N, 126°36′ E), and a total of 120 L *A. poporum* strain UTHC8 (hereafter named “North Sea”) isolated from the North Sea [22] were cultivated in aerated 10 L batch cultures in F/2 medium-Si [39] (for the Korean isolate) or in a half-strength modified K-medium [22] (for the North Sea isolate) in a growth chamber at 20 °C under an illumination of 20−50 µE·m^−2^·s^−1^ of cool white fluorescent light on a 14:10 h light-dark cycle. Cultures were harvested in late stationary phase at cell densities (irregularly checked by manual counting using an inverted microscope) ranging from about 1–3 × 10^5^ cell·mL^−1^. Cells in the culture flasks were lysed by addition of acetone (HPLC grade, J.T. Baker, Center Valley, PA, USA) to final concentration of 7%. One g·L^−1^ anion exchange resin (Diaion^®^ HP20, Supelco^®^, Steinheim, Germany), which previously had been preconditioned with methanol (HPLC grade, J.T. Baker, Center Valley, PA, USA) for 24 h, was added to the broth. After shaking for 24 h, the resin was then collected on a 100 µm Nitex mesh, dried, and frozen at −20 °C until extraction. For extraction, 300 g of HP20 resin were suspended in 400 mL of methanol and shaken for 2 h on a lab shaker and subsequently partitioned into two aliquots. Each aliquot was transferred into a glass chromatography column (3 cm diameter, 60 cm length) and was eluted with 500 mL methanol. The column elutes of each strain were reduced in a rotary evaporator, combined, and finally taken to dryness.

### 4.3. Purification of AZA-36 and AZA-37

The combined HP20 resin extracts were partitioned between ethyl acetate (150 mL) and aqueous NaCl (1 M, 50 mL). The ethyl acetate fraction was evaporated to dryness *in vacuo.* Approximately 2 g of silica gel was added to the dried extract. The sample was mixed to a fine powder and loaded on to a silica gel (6 g) column (6 × 4 cm). Vacuum-assisted elution was performed successively with hexane, ethyl acetate, ethyl acetate−methanol (9:1, 7:3, and 1:1), and methanol (30 mL of each, all containing 0.1% acetic acid except for hexane). The methanol fraction, which flow injection analysis (FIA)-MS/MS (method A) showed to contain the AZAs, was evaporated *in vacuo*, and the sample in acetonitrile–water (6:4, plus 0.1% triethylamine) was loaded onto a column packed with phenyl-hexyl (19.9 × 2 cm). The sample was eluted with acetonitrile–water (3:7, plus 0.1% triethylamine) at 4 mL·min^−1^, and 5 mL fractions were collected. Appropriate fractions were combined (fractions 18–25 for AZA-36 (**1**) and fractions 17–23 for (**2**)) based on FIA-MS/MS analysis.

Final purification was achieved by semi-preparative model 10AVp HPLC (Shimadzu, Kyoto, Japan) with photodiode array (PDA) detection (210 nm) using a Cosmosil C18 (5 µm, 250 × 4.6 mm, Nacalai tesque, Kyoto, Japan) column eluted with acetonitrile–water (2:3, plus 2 mM ammonium acetate) at 1 mL min^−1^. The column temperature was 30 °C. Purified (**1**) was recovered by evaporation to ~20% acetonitrile, loading on to a SPE cartridge (Oasis HLB, 200 mg), washing with methanol–water (1:9, 10 mL) to remove the buffer, and eluting with methanol–water (4:6, 6:4, 8:2, 10:0, 20 mL each) with >95% of the AZAs eluting in the 8:2 fraction. Removal of solvent by evaporation *in vacuo* afforded purified (**1**) and (**2**) as white solids.

### 4.4. LC-MS/MS Analysis

#### 4.4.1. Method A

Qualitative analysis of fractions for AZAs was performed by FIA-MS/MS using a Micromass (Manchester, UK) QTof Ultima. Samples (2 µL) were injected, using a Waters (Manchester, UK) model 2795 LC autosampler, directly (no column) into the mass spectrometer monitoring for the precursor ions: (**1**) *m*/*z* 858.5, and (**2**) *m*/*z* 846.5. The cone voltage was 40 V, the cone and desolvation gas flows were set at 100 and 800 L·h^−1^, respectively, and the source temperature was 150 °C.

#### 4.4.2. Method B

Recoveries were determined by quantitative analysis (against AZA-1 CRM) of fractions on a Waters 2795 LC coupled to a Micromass QTof Ultima operated in precursor ion scan mode monitoring for the following masses: AZA-1 *m*/*z* 842.5, (**1**) *m*/*z* 858.5, and (**2**) *m*/*z* 846.5. The MS parameters were the same as those described in method A.

An isocratic elution was used, with phase A consisting of water and phase B of 95% acetonitrile in water (both containing 2 mM ammonium formate and 50 mM formic acid). Chromatography was performed with an ACE C18 column (30 × 2.1 mm, 3 µm) (Advanced Chromatography Solutions, Aberdeen, UK). The injection volume was 5 µL and the column and sample temperatures were 25 °C and 6 °C, respectively.

#### 4.4.3. Method C

Purity was initially assessed on the QTof Ultima by running MS scans (*m*/*z* 100–1000) using binary gradient chromatographic conditions with phase A consisting of water and phase B of 95% acetonitrile in water (both containing 2 mM ammonium formate and 50 mM formic acid). Chromatography was performed with a Hypersil BDS C8 column (50 × 2.1 mm, 3 µm, with a 10 × 2.1 mm guard column of the same stationary phase) (Thermo Scientific, Waltham, MA, USA). The gradient was from 30% B, to 90% B over 8 min at 0.25 mL·min^−1^, held for 5 min, then held at 100% B at 0.4 mL min^−1^ for 5 min, and returned to the initial conditions and held for 4 min to equilibrate the system. The injection volume was 5 µL and the column and sample temperatures were 25 and 6 °C, respectively. Analysis for other known AZA analogues was also performed by product ion scans, where the precursor ions were selected and then fragmented.

#### 4.4.4. Method D

Purity was further assessed by UV analysis. A concentrated sample (~500 µg·mL^−1^) was injected (1 µL) onto the semi preparative system (model 10AVp, Shimadzu, Kyoto, Japan) with photodiode array (PDA) detection (210 nm) using a Cosmosil C18 column (5 µm, 250 × 4.6 mm) eluted with acetonitrile–water (2:3, plus 2 mM ammonium acetate) at 1 mL·min^−1^. The column temperature was 30 °C.

### 4.5. NMR Analysis

NMR spectra were recorded on an Avance 600 MHz NMR spectrometer (Bruker, Bremen, Germany) (600 MHz for ^1^H, 150 MHz for ^13^C) equipped with a cryo platform. All experiments were measured at 303 K. Compounds (**1**) and (**2**) were obtained as white powders and dissolved in methanol-*d*_4_ (3.31 ppm of residual CH_3_OH for ^1^H-NMR and 49.0 ppm for ^13^C-NMR). The spectra were all referred to solvent signals stated accordingly as internal standards.

### 4.6. Toxicity Assays

#### 4.6.1. Cell Culturing

Human Jurkat E6-1 T lymphocyte cells (American Type Culture Collection TIB-152; Manassas, VA, USA) were grown as previously described [31]. Briefly, cells were grown in RPMI-1640 medium (cat. #11875-093, Invitrogen, CA, USA) supplemented with 10% (v/v) fetal bovine serum (FBS; cat. #26140, Invitrogen, CA, USA) and maintained in a humidified incubator (18AIC-UV, Sanyo, Rutherford, NJ, USA) with 5%:95% CO_2_:air at 37 °C. Cells were subcultured with fresh medium at an inoculum ratio of 1:4 every 3 to 4 days by transferring 2.5 mL of cells to 7.5 mL of fresh supplemented medium in 75 cm^2^ screw cap culture flasks.

#### 4.6.2. Cytotoxicity Assay

To determine the effect of the AZA analogues on cellular toxicity, Jurkat T lymphocyte cells were continuously exposed to toxins and viability determined. Exponentially growing cells were seeded in a volume of 100 μL of the supplemented medium at a density of 30,000 cells per well in black, sterile, 96-well culture plates for 18–24 h to allow for recovery and settling. A range of final AZA concentrations were then added for 24, 48, or 72 h of continuous exposure prior to assessment of cytotoxicity. Parallel controls of equivalent amounts of methanol/PBS were used to normalize the viability data for each treatment. Cellular viability/cytotoxicity was assessed using the MTS (3-(4,5-dimethylthiazol-2-yl)-5-(3-carboxymethoxyphenyl)-2-(4-sulfophenyl)-2*H*-tetrazolium) assay (Promega Biosciences, San Luis Obispo, CA, USA; cat. no. G5421). Like other tetrazolium-based assays, MTS in the presence of an electron coupling reagent (phenazine methosulfate; PMS) measures cellular viability by determining the activity of mitochondrial dehydrogenases [40]. As a substrate for dehydrogenases, MTS becomes reduced into a soluble, purple dye that can be quantified colourimetrically to determine the relative level of cellular viability/cytotoxicity per well. Following exposure of the cells to the AZA analogues for a specified period of time, each well received 10 μL of a PMS/MTS (1:20) solution. Cells were incubated for 4 h after which absorbance readings at 485 nm were obtained using a FluoStar microplate reader (BMG Lab Technologies, Cary, NC, USA). Data are presented as means ± SE of three separate experiments (*n* = 3 biological replicates). In addition, within each experiment, every concentration of each AZA analogue was tested in duplicate wells. Cytotoxicity data were blank corrected and normalized to the control (% viability). EC_50_ and 95% confidence interval determinations were calculated using three parameter, variable slope, non-linear regression analysis (GraphPad Prism, ver. 5.0f, San Diego, CA, USA). All data (mean ± SE; *n* = 4) were normalized to the control (10% methanol vehicle). Non-linear, three parameter dose-response (variable slope) analysis was performed and EC_50_ values were calculated (Table 4).

## 5. Conclusions

AZP events to date have only occurred with mussels harvested in Europe, which is consistent with the occurrence of the AZA-producing species *A. spinosum* in the Northeast Atlantic. However, we now have evidence that other species such as *A. poporum* and *Amphidoma languida* are also AZA-producers [30]. Whereas *A. languida*, to date, has only been confirmed to occur in the Northeast Atlantic, we now know that *A. poporum* has a much wider distribution [22,28,29]. Our data suggest that there is a potential risk of AZP in the North West Pacific area. The lower toxicity of AZA-36 is probably not the only reason for the lack of AZP events in the west Pacific, but environmental factors that control growth and proliferation of the producing species most likely also play an important role [41]. For this reason we suggest that AZA-36 and AZA-37 should be included in shellfish safety monitoring programmes. Currently both compounds can only be quantified as AZA-1 equivalents until standards for these compounds are made available. Although *in vitro* AZA-36 and AZA-37 are 6- and 3- fold less toxic than AZA-1, respectively, knowledge of the presence of these toxins in seafood, especially if associated with the presence of other shellfish toxins, is important for the health of shellfish consumers. Certainly more research is required to reveal the relationship between the occurrence of AZA-producers and AZP events.

## Supplementary Files

Supplementary File 1
