# Supplementary material for: Structure Elucidation and in Vitro Toxicity of New Azaspiracids Isolated from the Marine Dinoflagellate Azadinium poporum
Source: Mar Drugs. 2015 Oct 30;13(11):6687–702. doi: 10.3390/md13116687 (PMC4663548; doi:10.3390/md13116687)
Supplement: Supplementary File 1 [file marinedrugs-13-06687-s001.docx]

**Supporting Information**

Structural elucidation of new azaspiracids isolated from *Azadinium poporum*


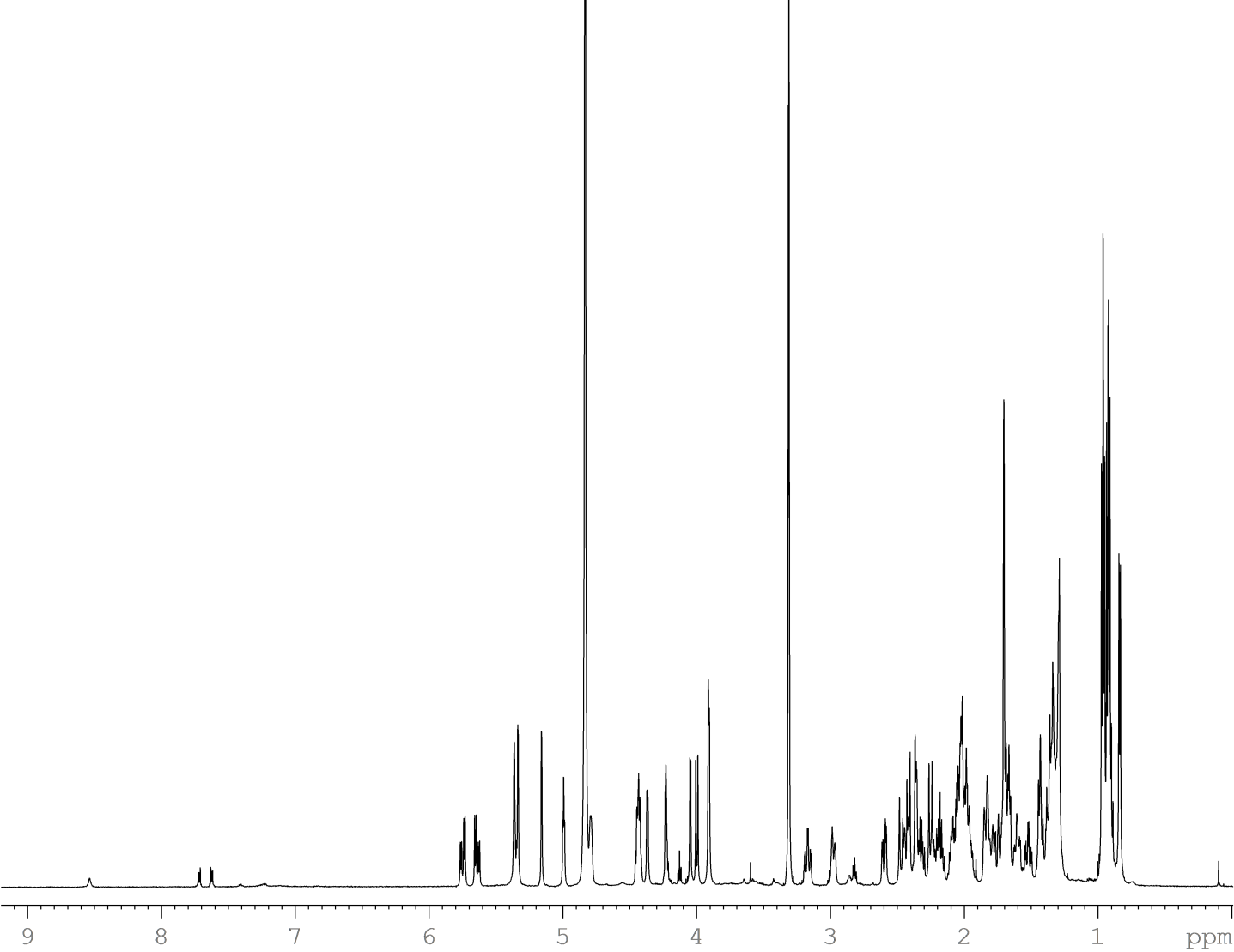


**Figure S1.** 1D ^1^H-NMR spectrum of AZA-36 (**1**) in CD_3_OD, 303 K, 600 MHz


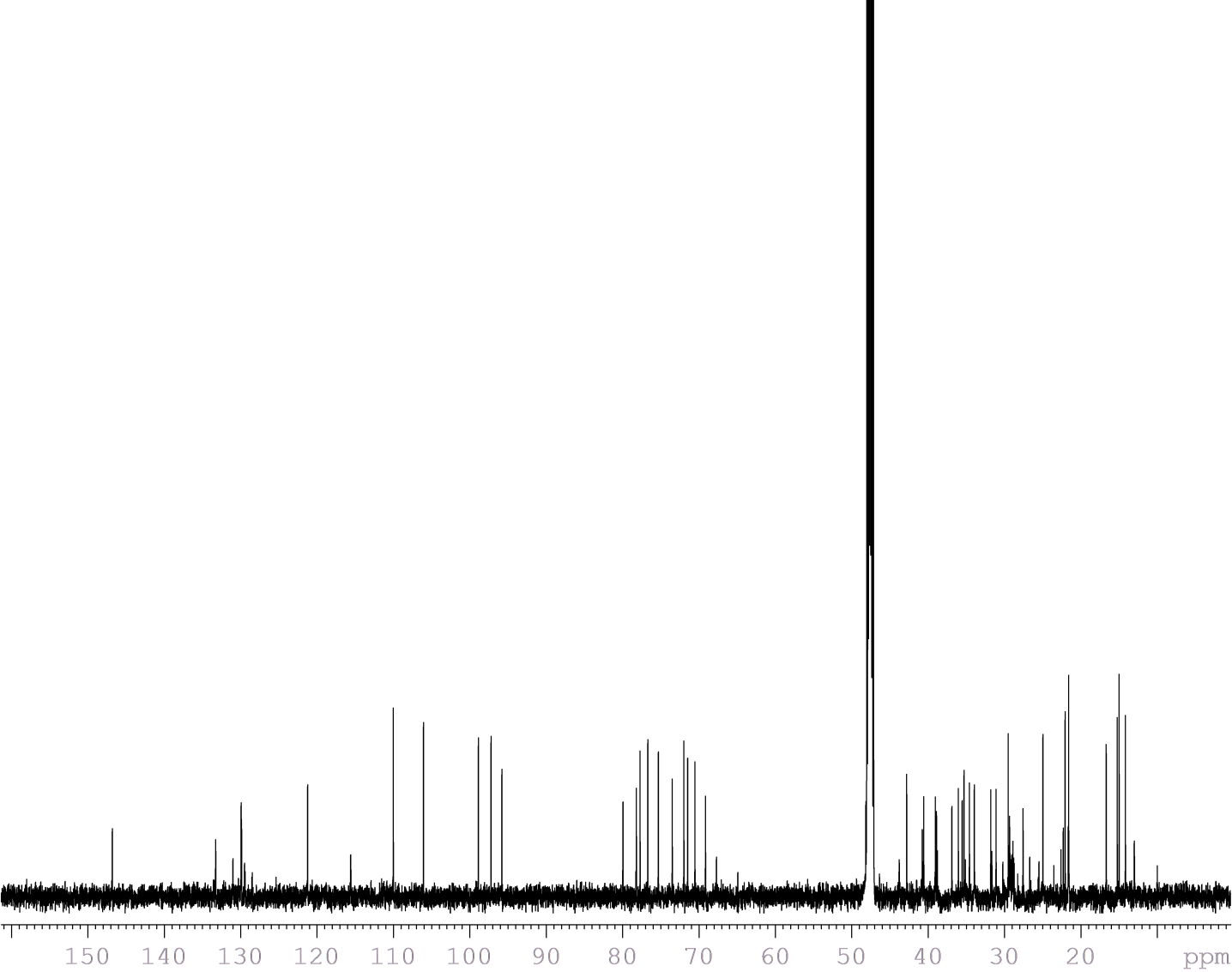


**Figure S2.** 1D ^13^C-NMR spectrum of AZA-36 (**1**) in CD_3_OD, 303 K, 150 MHz


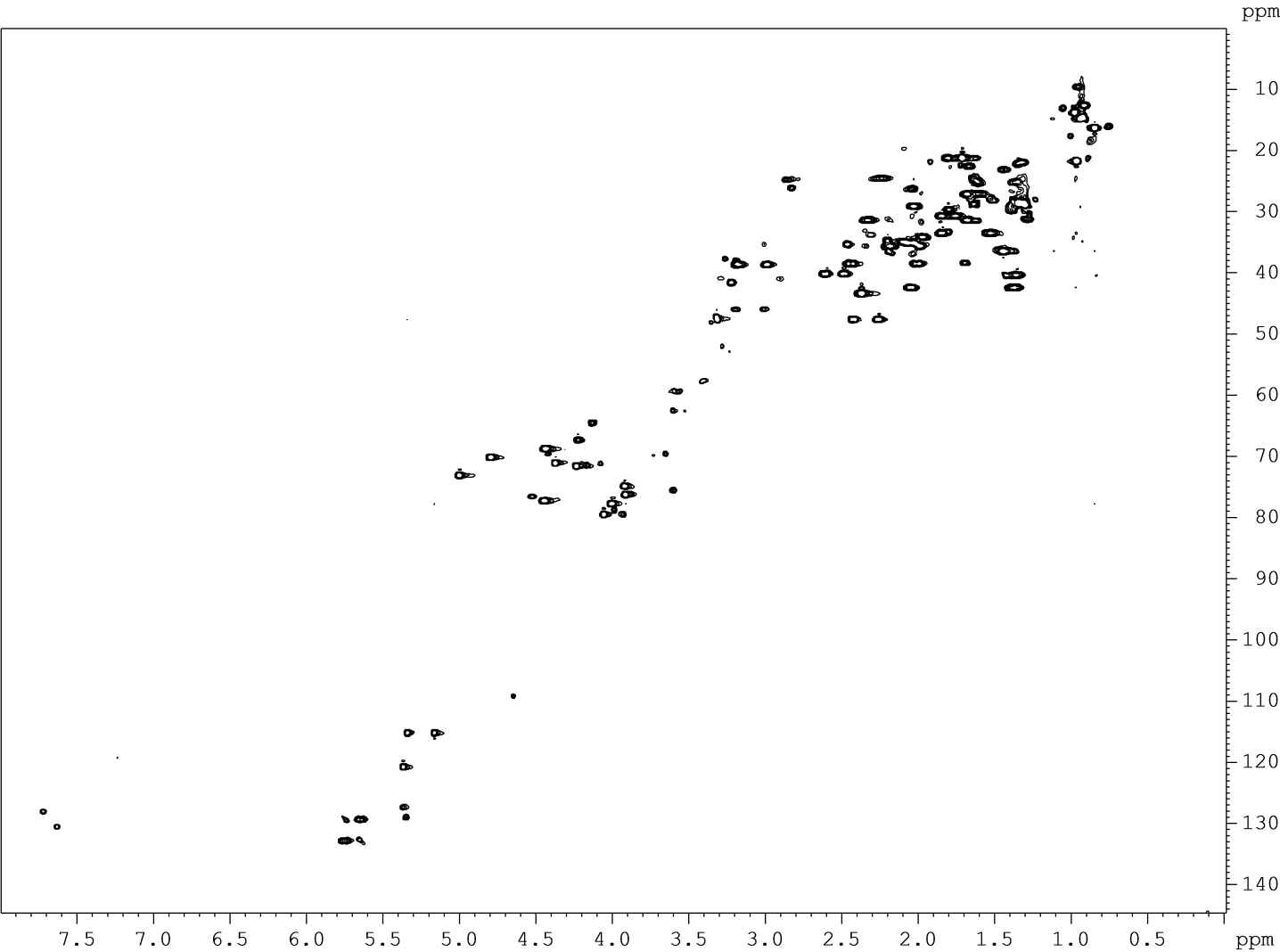


**Figure S3.** 2D ^1^H,^13^C-HSQC spectrum of AZA-36 (**1**) in CD_3_OD, 303 K


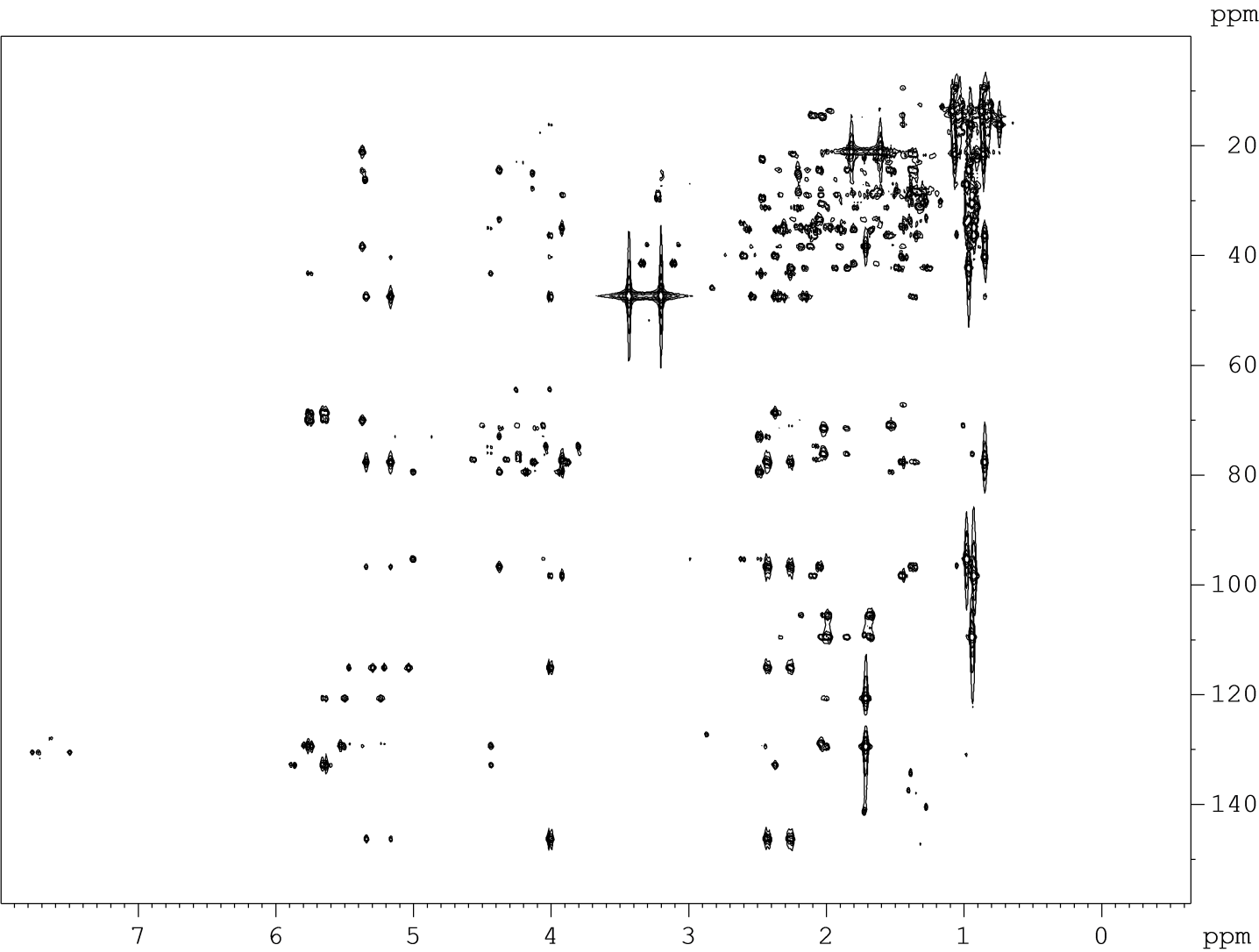


**Figure S4.** 2D ^1^H,^13^C-HMBC spectrum of AZA-36 (**1**) in CD_3_OD, 303 K


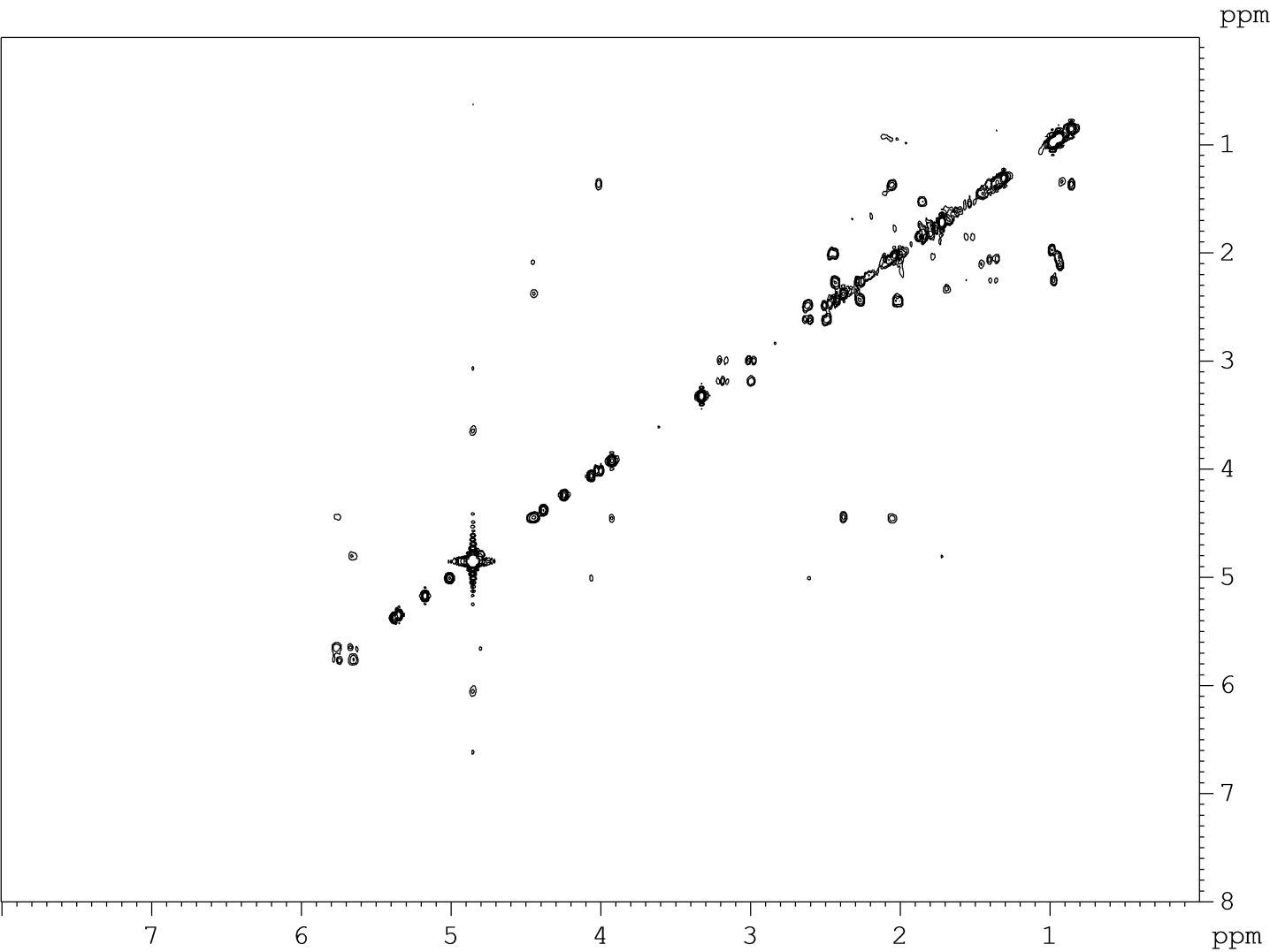


**Figure S5.** 2D ^1^H,^1^H-COSY spectrum of AZA-36 (**1**) in CD_3_OD, 303 K


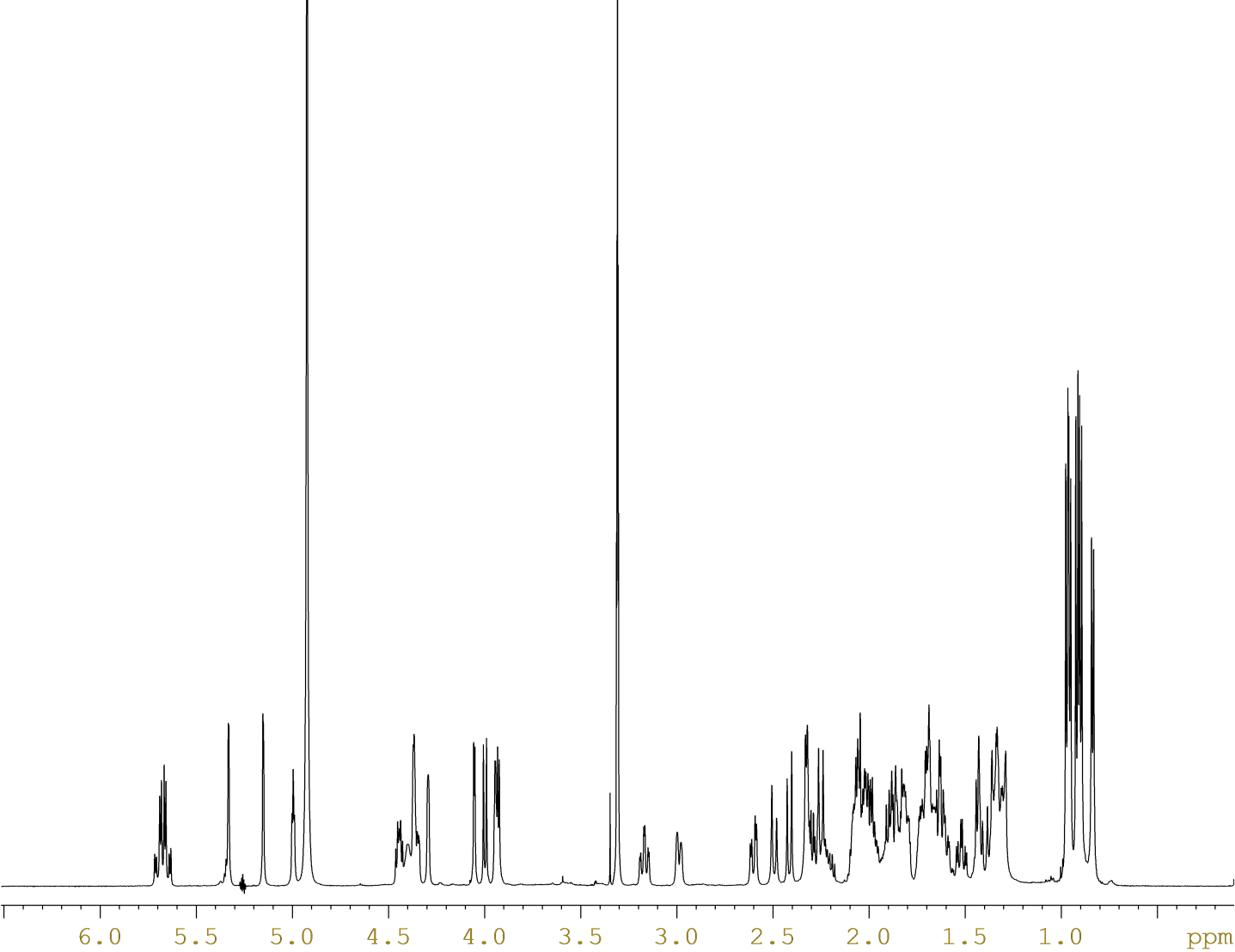


**Figure S6.** 1D ^1^H-NMR spectrum of AZA-37 (**2**) in CD_3_OD, 303 K, 600MHz


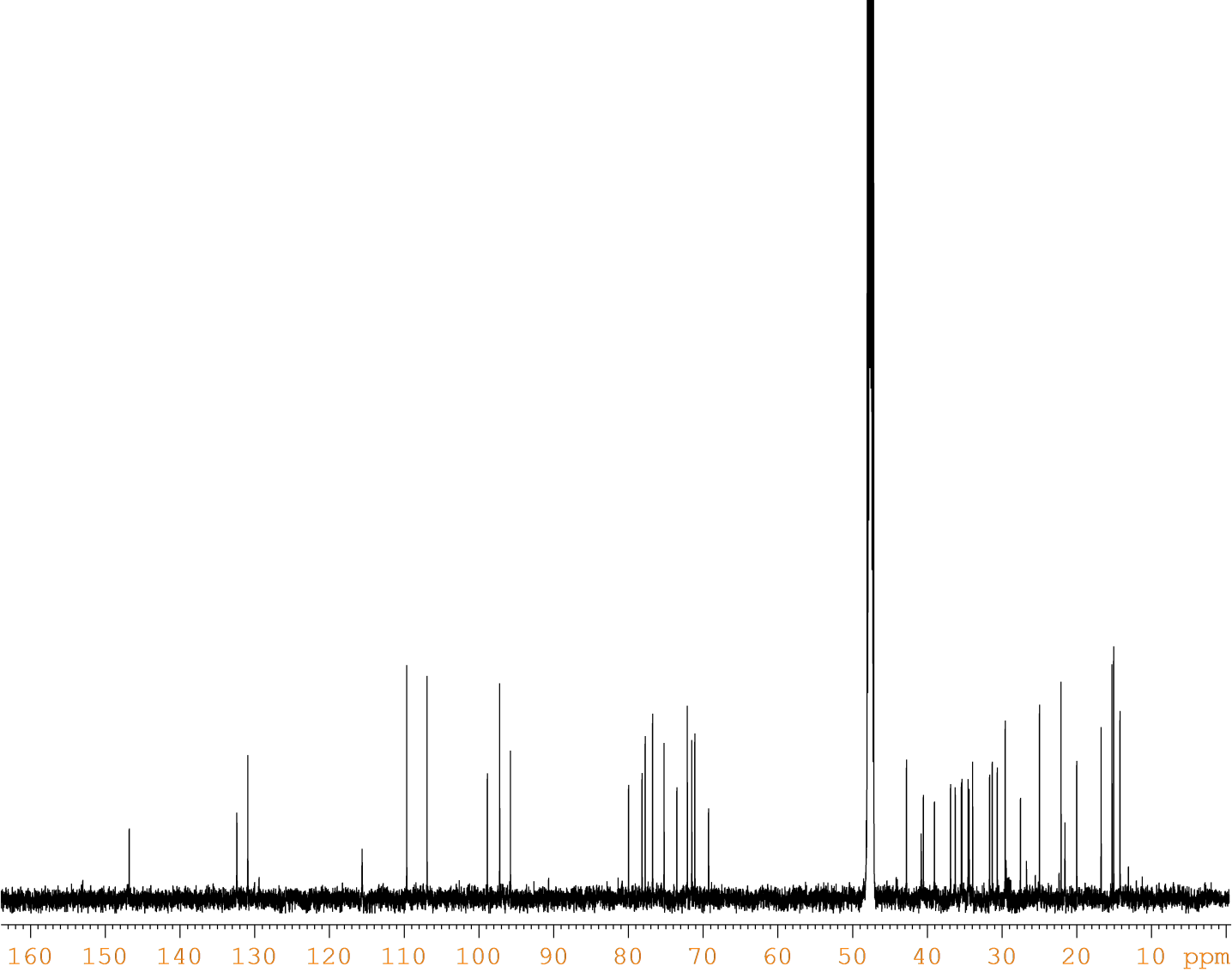


**Figure S7.** 1D ^13^C-NMR spectrum of AZA-37 (**2**) in CD_3_OD, 303 K, 150 MHz


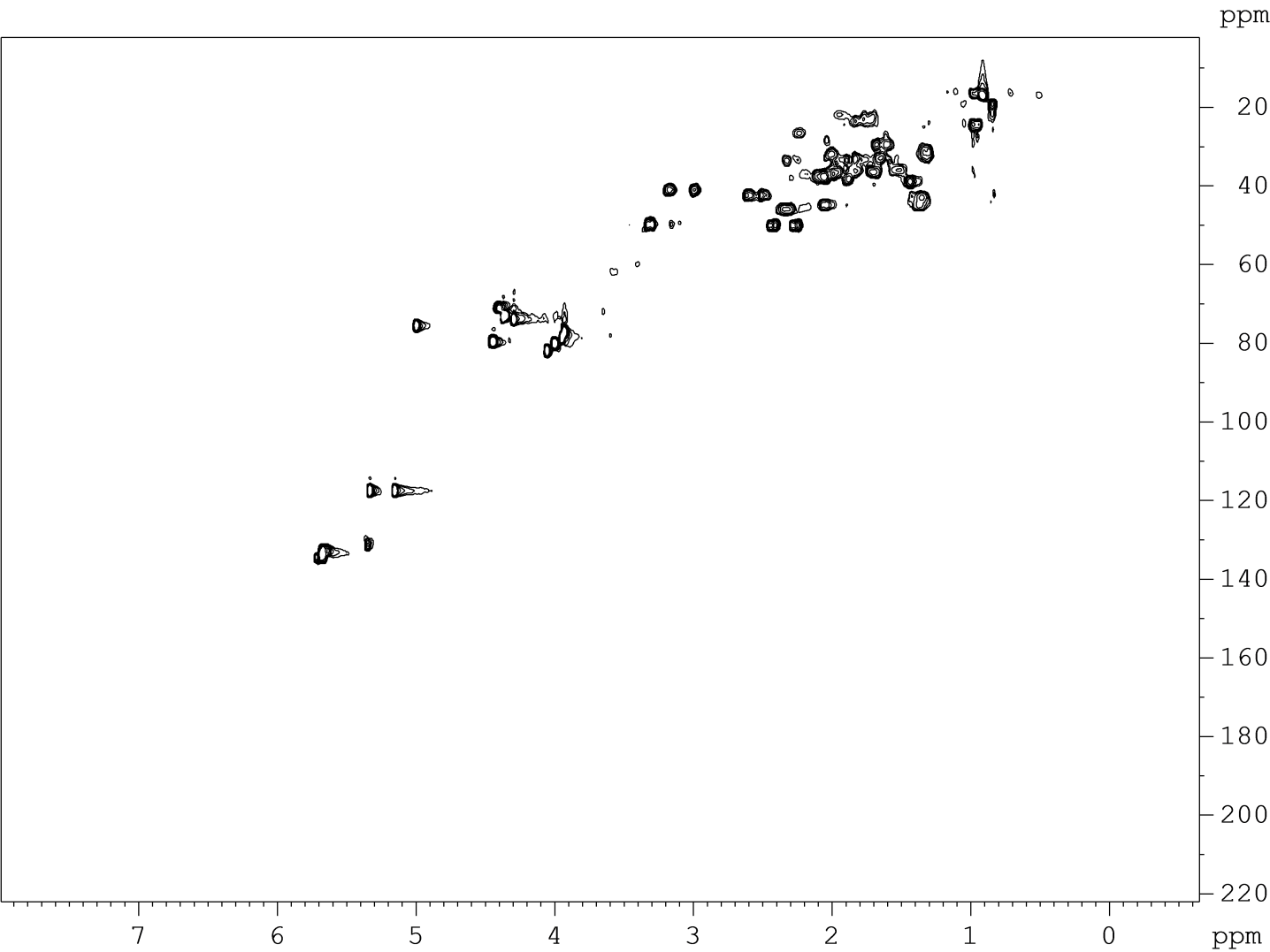


**Figure S8.** 2D ^1^H,^13^C-HSQC spectrum of AZA-37 (**2**) in CD_3_OD, 303 K


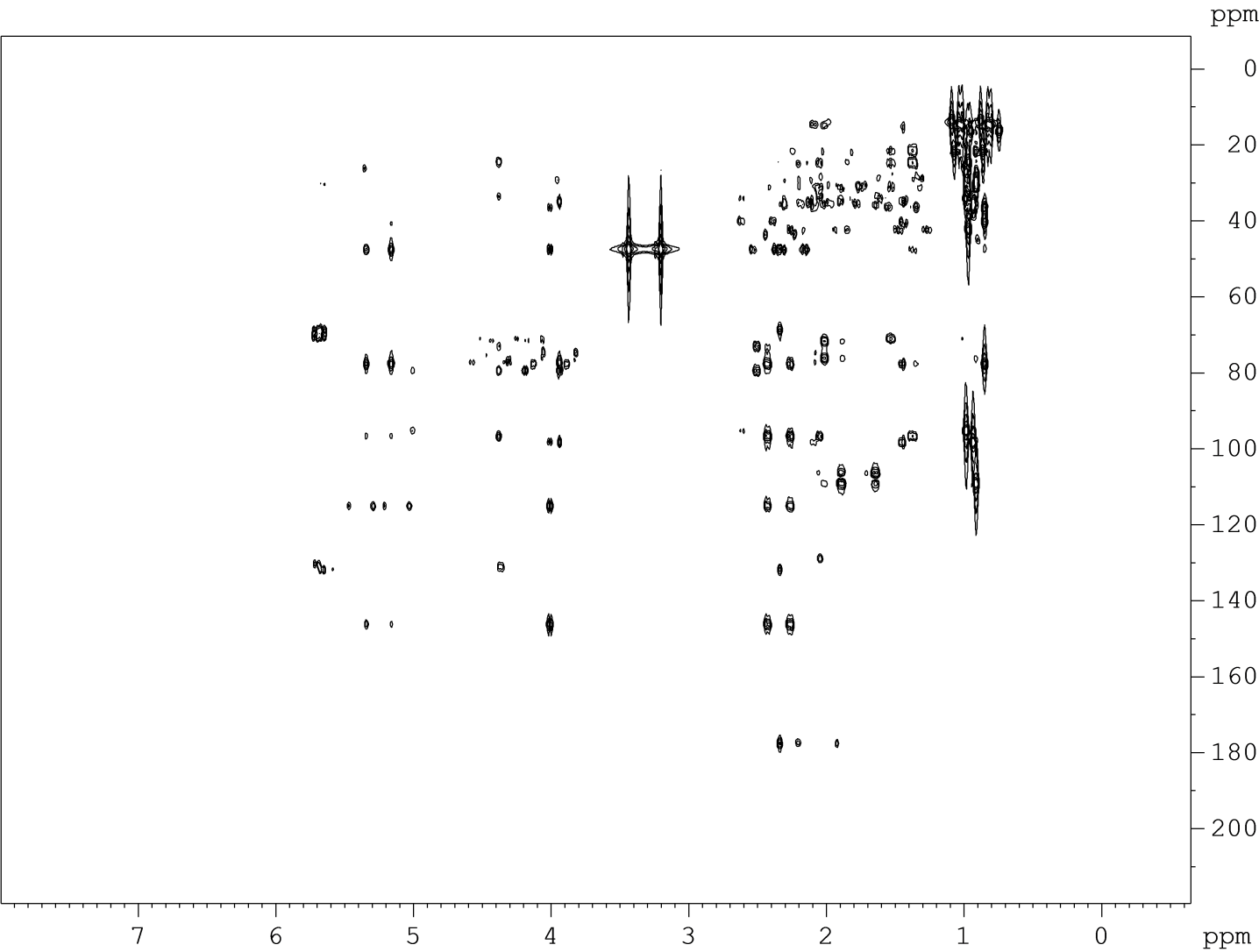


**Figure S9.** 2D ^1^H,^13^C-HMBC spectrum of AZA-37 (**2**) in CD_3_OD, 303 K


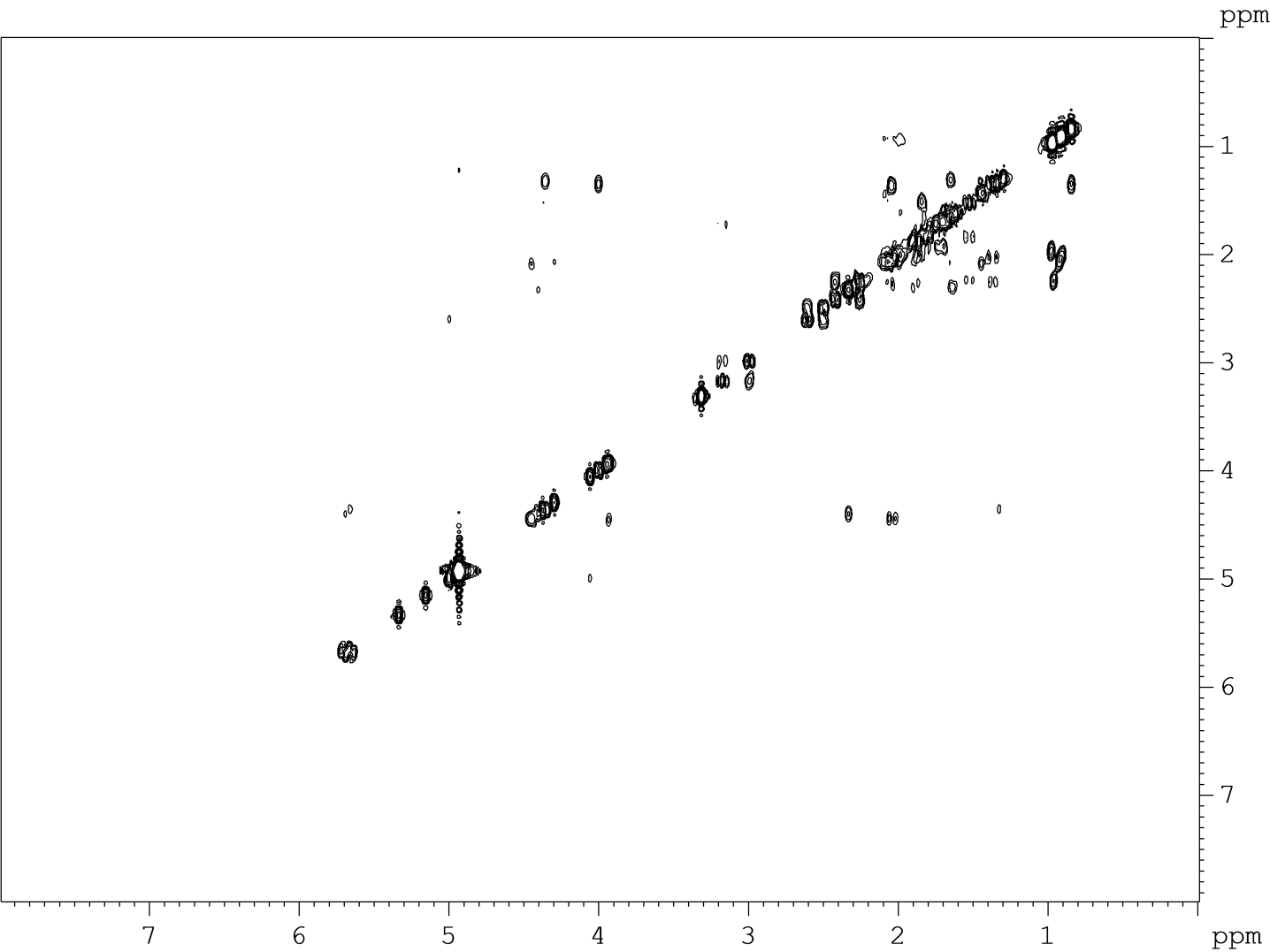


**Figure S10.** 2D ^1^H,^1^H-COSY spectrum of AZA-37 (**2**) in CD_3_OD, 303 K S7
